# Spark Plasma Sintering-Assisted Synthesis of Bi_2_Fe_4_O_9_/Bi_25_FeO_40_ Heterostructures with Enhanced Photocatalytic Activity for Removal of Antibiotics

**DOI:** 10.3390/ijms232012652

**Published:** 2022-10-21

**Authors:** Zhifei Liu, Yaqi Tan, Xuefeng Ruan, Jing Guo, Wei Li, Jiajun Li, Hongyu Ma, Rui Xiong, Jianhong Wei

**Affiliations:** Key Laboratory of Artificial Micro- and Nano-Structures of Ministry of Education, School of Physics and Technology, Wuhan University, Luojiashan Road, Wuhan 430072, China

**Keywords:** bismuth ferrite, spark plasma sintering, heterostructure, visible-light irradaition, antibiotics

## Abstract

Bismuth ferrite-based heterojunction composites have been considered as promising visible-light responsive photocatalysts because of their narrow band gap structure; however, the synthetic methods reported in the literature were usually time-consuming. In this study, we report a facile and quick preparation of bismuth ferrite-based composites by the hydrothermal method, combined with spark plasma sintering (SPS), a technique that is usually used for the high-speed consolidation of powders. The result demonstrated that the SPS-assisted synthesized samples possess significant enhanced photoelectric and photocatalytic performance. Specifically, the SPS650 (sintered at the 650 °C for 5 min by SPS) exhibits a 1.5 times enhancement in the photocurrent density and a 3.8 times enhancement in the tetracycline hydrochloride photodegradation activity than the unmodified bismuth ferrite samples. The possible influence factors of SPS on photoelectric and photocatalytic performance of bismuth ferrite-based composites were discussed carefully. This study provides a feasible method for the facile and quick synthesis of a highly active bismuth ferrite-based visible-light-driven photocatalyst for practical applications.

## 1. Introduction

Over the past few decades, the development of sustainable energy resources, such as solar energy, wind energy, hydrogen energy, and geothermal energy, etc., has become one of the most important issues around the world because of the shortage of conventional energy resources and environmental pollution problem. Among various sustainable energies, solar energy has attracted much attention, due to its inexhaustibility, environmental friendliness, and wide availability, etc. [1,2]. Semiconductor photocatalytic technology, which can directly convert solar energy into chemical energy through semiconductor photo-catalysts, has been recognized as one of the most attractive solutions for achieving sustainable energy supply and environmental restoration [3,4,5,6].

The common semiconductor photocatalytic materials include TiO_2_, ZnO, SrTiO_3_, Bi_2_S_3_, GaN, etc. Compared with the other photocatalytic materials, TiO_2_ possesses the advantages of stable, non-toxic, high activity, photo-corrosion resistance, etc., and these characteristics make it one of the most representative semiconductor photocatalysts. However, the low utilization of visible light and high electron-hole recombination rate limit its wide application. In order to expand the spectral absorption range of TiO_2_ photocatalyst and improve its photocatalytic efficiency, scientists have tried a variety of methods, such as metal/non-metal doping, hetero-junction construction, dye sensitization, etc. [7,8,9,10]. On the other hand, much effort had been paid to the development of novel, visible-light-responsive photocatalytic materials, such as metal oxides, metal sulfide, metal nitride, and organic compounds, etc. Recently, bismuth ferrite (BFO), such as BiFeO_3_, Bi_2_Fe_4_O_9_, Bi_25_FeO_40_, etc., have attracted much attention as a kind of potential candidate for visible-light responsive photocatalysts because of their narrower band gap structure (1.5~2.2 eV) and ability to degrade organic pollutants under visible light illumination [11,12,13,14,15]. However, there are still two issues, such as fast electron-hole recombination and low quantum yield, that limit its practical application. To further improve their photocatalytic performance, much effort had been made, such as synthesis with different methods and the control of process parameters, etc. However, most synthetic methods reported in the literature are time-consuming. Thus, developing a more economical and facile method to obtain highly efficient BFO-based photocatalyst is desirable.

Spark plasma sintering (SPS) is a typical field-assisted sintering technique, which is usually used for a high-speed consolidation of powders [16,17,18]. Recently, M.A. Lange reported that the heat treatment of WO_3−x_F_x_ by SPS on a minute (<10 min) scale can obtain higher photocatalytic performance [19]. Yang reported that self-doping TiO_2_ with the use of directly treated commercial P25 at a desired temperature for only 5 min through SPS technology exhibited significantly high photoelectric and photocatalytic performance [20].

Compared with the other high-temperature heat treatment technologies, SPS possesses the following advantages. Firstly, its temperature can be raised quickly in a short time, while conventional methods usually take much longer to reach the same temperature. Secondly, the direct pulse current applied in the SPS process tends to activate the surface of the particles, thereby generating a large amount of hydroxyl radicals and atomic oxygen or oxygen-free radicals, which is profitable for the promotion of photocatalytic activity. Although the effects of SPS heat treatment on the ferroelectric, piezoelectric, and dielectric properties of bismuth ferrite (BFO) materials have been reported [21,22,23], the effect of SPS heat treatment on the photoelectric and photocatalytic activity of BFO materials is still lacking.

**Figure 1:**
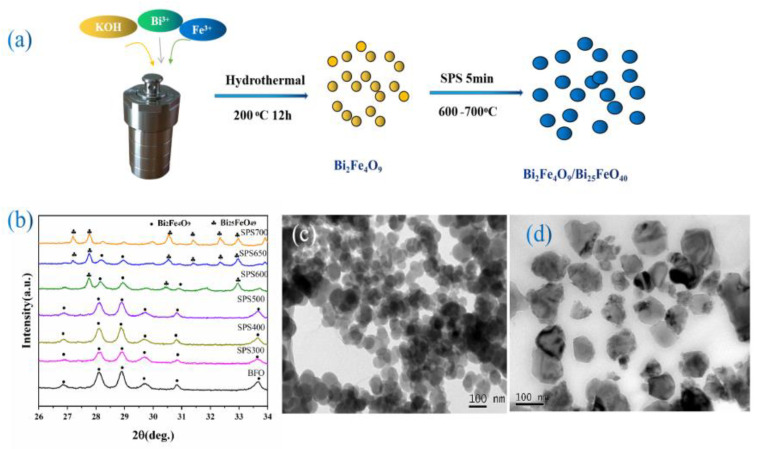
(**a**) Preparation scheme for bismuth ferrites, (**b**) X-ray diffraction (XRD) spectra of as-prepared samples, (**c**) SEM image of untreated BFO, (**d**) SEM image of SPS650.

To explore the influence of SPS on the BFO photocatalysts and expand its application range, in this study, we reported a facile and rapid preparation of BFO-based composites by the direct use of hydrothermal-prepared BFO at a desired SPS temperature X (X = 300, 400, 500, 600, 650, 700) °C for only 5 min, and the corresponding products were named SPSX. The overall procedure for the synthesis of heterostructured BFO-based composites is shown in Figure 1a.

## 2. Results and Discussion

The phase structure of the as-prepared samples was determined by X-ray diffraction (XRD) (Figure 1b and Figure S1). As shown, the unmodified sample (BFO) and low-temperature SPS treatment samples (SPS300, SPS400, and SPS500) exhibited similar diffraction patterns, all diffraction peak patterns can be indexed to orthorhombic Bi_2_Fe_4_O_9_ (JCPDS#25-0090) [24,25,26], and no other impurity phase appeared, indicating that low-temperature SPS treatment has no obvious effect on its phase structure. When the SPS heating temperature increased to 600 °C, two strong diffraction peaks at 28.2° and 28.9°, corresponding to the (121) and (211) crystal planes of orthorhombic Bi_2_Fe_4_O_9_, weakened, and two new characteristic peaks appeared at 27.8° and 32.9°, which can be attributed to the (310) and (321) crystal planes of cubic Bi_25_FeO_40_, according to the PDF standard cards (JCPDS#46-0416) [27,28], which means that the Bi_25_FeO_40_ and Bi_2_Fe_4_O_9_ phase coexistence was at 600 °C. With the increasing of the SPS processing temperature (SPS 650), the content of Bi_25_FeO_40_ phase gradually increased. At 700 °C, Bi_2_Fe_4_O_9_ was almost entirely transformed into Bi_25_FeO_40_. Anyway, the sample treated at 300, 400, and 500 °C under SPS for 5 min exhibited similar photocatalytic activity as the unmodified BFO (omitted here). For the sake of simplicity, in this work, we mainly reported the unmodified BFO sample and the samples treated at 600, 650, and 700 °C under SPS for 5 min and the percentage of Bi_2_Fe_4_O_9_ and Bi_25_FeO_40_ in the BFO, SPS600, SPS650, SPS700 were shown in Table S1. The morphology of the BFO and SPS650 samples was observed by scanning electron microscope (SEM) (Figure 1c,d). As indicated in Figure 1c, the unmodified BFO particles are near spherical with average diameter of ca. 70 nm. After being treated by SPS 5 min at 650 °C, the shape of BFO became irregularly spherical, with an average diameter reaching 80–90 nm (Figure 1d, Figures S2 and S3). In Figure S4, the peaks of Bi 4f, Fe 2p, and O 1s can be observed in the survey XPS spectra of the BFO samples before and after SPS modification, indicating that the elements contained in the sample were consistent with the target product.

**Figure 2:**
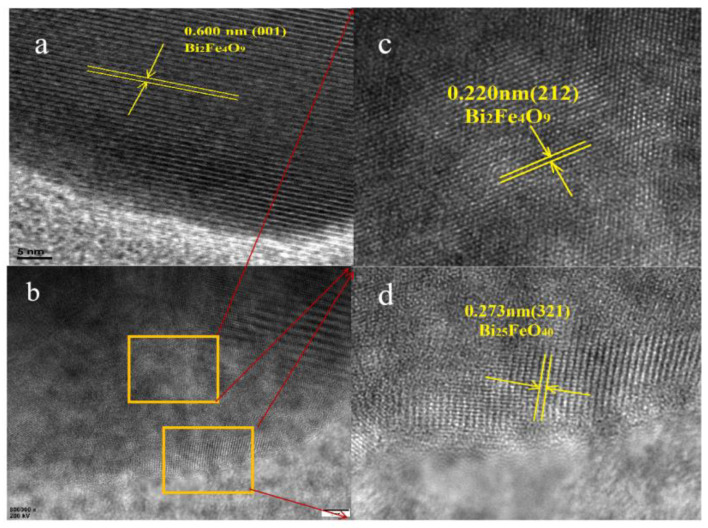
HRTEM image of (**a**) untreated BFO, (**b**) SPS650, (**c**,**d**) corresponding magnified image of (**d**).

For clarity, the HRTEM of as-prepared BFO and SPS650 samples were performed to further elucidate the microstructure of the samples. For unmodified BFO (Figure 2a), the lattice fringes of the (001) planes with interplanar spacing of approximately 0.600 nm corresponded to Bi_2_Fe_4_O_9_ [29,30]. For SPS650 (Figure 2b–d), the fringe of the (212) plane with interplanar spacing of approximately 0.220 nm corresponded to Bi_2_Fe_4_O_9_, while the fringes of the (321) planes with interplanar spacing of approximately 0.273 nm corresponded to Bi_25_FeO_40_ [31,32,33], indicating some of Bi_2_Fe_4_O_9_ transformed into Bi_25_FeO_40_ when treated with SPS for 5 min, and the Bi_2_Fe_4_O_9_/Bi_25_FeO_40_ heterojunction was synchronously formed during this process.

**Figure 3:**
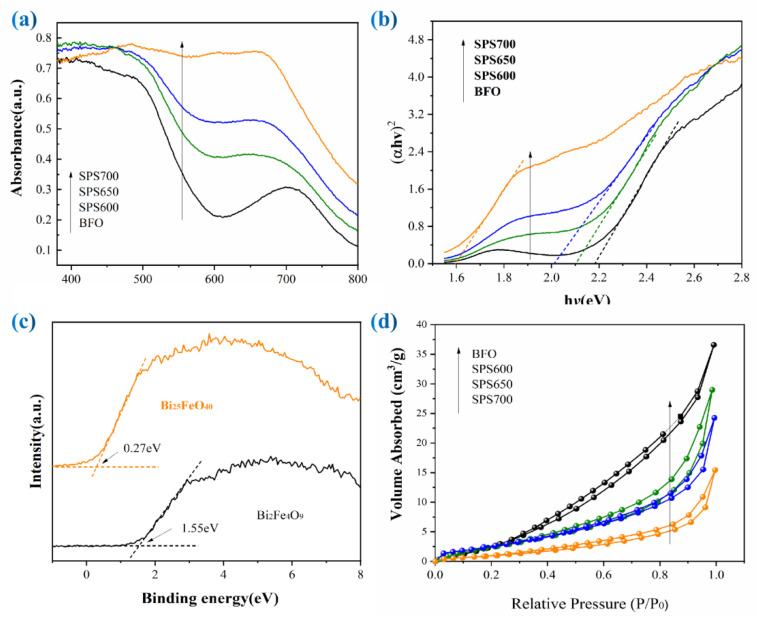
(**a**) The UV–Vis absorption spectrum of the as-prepared sample; (**b**) the calculation bandgap diagram of the as-prepared sample, according to Kubelka–Munk function. (**c**) The XPS VB spectra of as-synthesized Bi_2_Fe_4_O_9_ and Bi_25_FeO_40_ samples; (**d**) N_2_ adsorption and desorption isotherms of the as-prepared samples.

The optical absorption properties of the as-prepared samples were investigated by UV–Vis DRS technology, as shown in Figure 3a,b. As indicated, all the samples can response to visible light; however, in comparison with the unmodified BFO, the samples with SPS treatment exhibited a significant enhancement in absorption intensities, along with the absorption redshift. The absorption edges of BFO located at ~570 nm corresponds to an optical bandgap (*Eg*) of 2.18 eV, which was calculated from the tangent line in the plot of the K-M function ${\left(\alpha hv\right)}^{2}$ vs. photo energy (*hv*) by extrapolating the tangent line to another small absorption edge at ~810 nm, which corresponds to an optical band gap of 1.60 eV. The absorption edge at ~570 nm can be ascribed to the electronic transfer from the valence band to the conduction band, and the absorption at ~810 nm is ascribed to the d-d transitions of Fe [34,35,36]. For the Bi_2_Fe_4_O_9_/Bi_25_FeO_40_ samples, the absorption edge of SPS700 was ~775 nm, and the absorption edges of SPS600 and SPS650 lied somewhere in between. According to Kubelka–Munk function, the band gaps for SPS600, SPS650, and SPS700 were 2.10 eV, 2.02 eV, and 1.60 eV, respectively (Figure 3b). In view of the preparation process, the close contact and strong interaction between the Bi_2_Fe_4_O_9_ and Bi_25_FeO_40_ existence in the SPS-treated samples is suggested. The valence band position (VB) of semiconductors was further determined by X-ray photoelectron spectroscopy, as shown in Figure 3c, which indicates that the VB (Ev) of Bi_2_Fe_4_O_9_ (BFO) and Bi_25_FeO_40_ (SPS700) were about 1.55 eV and 0.27 eV, respectively. Correspondingly, the conductor band positions (CB, Ec) of Bi_2_Fe_4_O_9_ (BFO) and Bi_25_FeO_40_ (SPS700) were estimated to be −0.63 eV and −1.33 eV, respectively, according to the equation: Ec = Eg − Ev.

The nitrogen adsorption isotherms (S_BET_) of the as-prepared catalysts were shown in Figure 3d, and the specific surface area (S_BET_) was calculated according to the Brunauer–Emmet–Teller method. The order of S_BET_ was as follows: SPS650 (23.1 m^2^∙g^−1^) > SPS600 (19.2 m^2^∙g^−1^) > SPS700 (15.8 m^2^∙g^−1^) > BFO (11.7 m^2^∙g^−1^). In our experiment, with the increasing of heat-treated temperature, the Bi_2_Fe_4_O_9_ phase gradually changed into the Bi_25_FeO_40_ phase. The specific surface area is connected to the crystal structure and particle stacking way, so the SPS650 sample exhibited the optimum specific surface area in our case. A larger surface, in general, means more surface-active sites and faster interfacial charge transfer for the reaction; thus, the SPS650 sample was expected to exhibit the higher photocatalytic activity.

**Figure 4:**
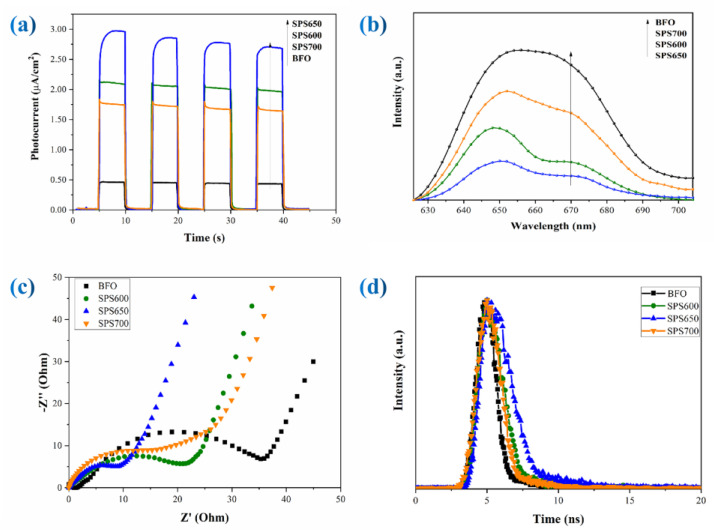
(**a**) The curve of photocurrent density versus time; (**b**) photoluminescence spectrum (PL), (**c**) electrochemical impedance spectroscopy (EIS), (**d**) transient photoluminescence spectrum (TRPL).

The separation and transfer rate of photogenerated electrons and holes are also the main factors affecting the photocatalytic performance. Herein, to explore the photoelectric separation and transferring performance of the as-prepared samples, the photocurrent response (I-t), photoluminescence emission spectra (PL), electrochemical impedance spectroscopy (EIS), and transient photoluminescence spectra of the as-obtained samples were carefully explored (Figure 4). Generally, the transient photocurrent reflects the charge carrier density and charge mobility. The stronger the photocurrent, the greater the density of the photo-generated carrier, and the more efficiencies of the charge separation [37,38,39,40]. As indicated in Figure 4a, the SPS650 sample exhibited the highest photocurrent density of 2.78 μA/cm^2^, which is about 5.56 times that of unmodified BFO (0.5 μA/cm^2^), and far larger than that of SPS600 (2.20 μA/cm^2^) and SPS700 (1.75 μA/cm^2^).

Figure 4b indicates the PL spectra of the as-prepared samples at an excitation wavelength of 325 nm. In general, PL emission intensity is related to the recombination of excited electrons and holes—the lower the PL peak intensity, the smaller the probability of recombination [41,42]. As indicated, the PL intensity of the SPS650 sample is the weakest among all the samples, inferring that the recombination rate of charge carries is the smallest among all the samples. The possible reason is maybe due to the fact that the moderate heterogeneous junctions produced in the SPS650 sample effectively accelerate the charge transfer and correspondingly reduces the charge’s recombination. As a result, the problem of fast charge recombination, as a historical intrinsic drawback of BFO photocatalysts, was effectively restrained by simply modifying the BFO by SPS heat-treatment for 5 min at a set temperature. Additionally, the diameters of the arc radius on the EIS Nynquist plot of the SPS-treated BFO samples were smaller to that of the unmodified BFO, while the SPS650 sample shows the least arc radius (Figure 4c). The smaller the internal resistance of the charge transfer means the higher the migration rate of the photo-generated electron-hole pairs and the higher the carrier separation rate [43,44,45]. Anyway, the time-resolved PL (TRPL) measurement results of the as-prepared samples indicate that the SPS650 composites have longer lifetimes than all the other samples (Figure 4d). In other words, compared with the other samples, the photoinduced electron-hole pairs in SPS650 are easier separated and transferred to the sample surface through an interfacial interaction between two different bismuth oxides, correspondingly resulting in a higher photocatalytic performance.

**Figure 5:**
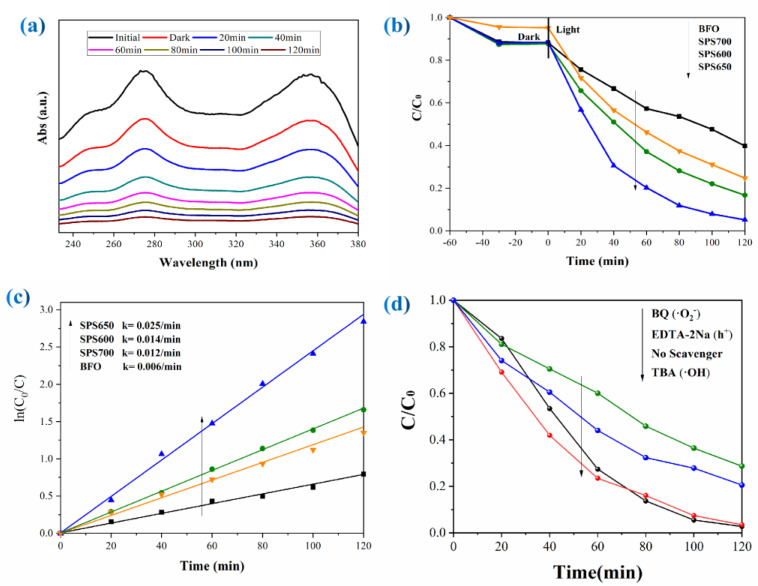
(**a**) Absorption spectra of the TCH solution over SPS650 under visible light irradiation; (**b**) photocatalytic degradation of TCH under visible light irradiation for as-prepared samples; (**c**) the corresponding photodegradation kinetics curves for as-prepared samples; (**d**) photocatalytic activities over the SPS650 sample with different scavengers for the degradation of TCH under visible-light irradiation.

Tetracycline hydrochloride (TCH) is a commonly used, but difficult to self-degrade, antibiotic [46]. Therefore, the photocatalytic performance of the as-prepared samples was evaluated by TCH as the target degradation product under visible light irradiation. As shown in Figure 5a, the characteristic absorption peaks at 275 nm and 356 nm decreased with the increasing of irradiation time, indicating the photodegradation of TCH under visible-light irradiation in the presence of catalysts. Figure 5b indicates that about 58% of TCH are degraded by BFO for 2 h, while the SPS-treated samples exhibited significantly enhanced photocatalytic performance, and the degradation ratios of TCH reached 82%, 96%, and 74% for the SPS600, SPS650, and SPS700 samples, respectively. It has been found that the SPS650 sample demonstrates the optimum photocatalytic activity, revealing the synergistic effect between Bi_25_FeO_40_ and Bi_2_Fe_4_O_9_, which is beneficial to the separation of photo-generated carriers, correspondingly resulting in a higher photocatalytic performance. This value is also higher than some previously reported catalysts (Table S3)[47,48,49,50,51,52]. The linear plots of (C_0_/C) versus irradiation time (t) suggest a pseudo-first order kinetic (Figure 5c). The rate constants k are estimated to be 0.006 min^−1^, 0.014 min^−1^, 0.025 min^−1^, and 0.012 min^−1^ for the samples BFO, SPS600, SPS650, and SPS700, respectively. The apparent quantum yield (*AQY*%) was calculated to be 4.66%, according to equation [47,48], and the detailed calculation can be found in Table S2:

$$AQY\%=\frac{\mathrm{the}\;\mathrm{number}\;\mathrm{of}\;\mathrm{degraded}\;\mathrm{moleculars}}{N}\times 100\%$$

Obviously, with the increasing of SPS heat-treat temperature, the photocatalytic efficiencies of the composites firstly increase to a maximal value and then decrease. The photo-reactive rate constant of the sample SPS650 was at 0.025 min^−1^, which is about 4.2 times that of unmodified BFO, 1.8 times that of the sample SPS600, and 2.1 times that of the sample SPS700. The reason for the relatively weaker photocatalytic performance for SPS600 and SPS700 can be ascribed to the fact that the amount of Bi_2_Fe_4_O_9_/Bi_25_FeO_40_ heterojunction was not formed enough. In addition, the photodegradation efficiency of SPS650 had slightly decreased after 5 cycle times (Figure S5), inferring its excellent stability, which is beneficial for the practice application.

The total organic carbon (TOC) removal ratio was used to evaluate the mineralization efficiency of TCH by SPS650 under visible light irradiation. As shown in Figure S6, the TOC decreased gradually, and the efficiency of TOC removal was 67% under 2 h irradiation. It is worth noting that the efficiency of TOC removal was lower than that of photodegradation, which can be attributed to the incomplete mineralization of the TCH or generation of a low molecular weight organic.

To confirm the speculation about the main active species, the agents of 1,4-benzoquinone (BQ, 0.1 mmol), disodium ethylenediamine tetraacetate (EDTA-2Na, 0.1 mmol), and tert-butyl alcohol (TBA, 0.1 mmol) were introduced as the superoxide radical (•O^2−^) scavenger, hole (h^+^) scavenger, and hydroxyl radical (•OH) scavenger, respectively. As indicated in Figure 5d, the degradation ratio of TCH over SPS650, without adding scavengers, was 96%. With the adding of BQ and EDTA-2Na, the degradation efficiencies of TCH were reduced to 63%, 72%, and 96%, respectively, indicating that superoxide radical was main active species, and the hole plays a relatively important role in the photocatalytic oxidation process. On the contrary, the photocatalytic activity of SPS650 had no obvious change by the addition of TBA, which infers that the hydroxyl radical was not the main active species to the degradation process.

**Scheme 1:**
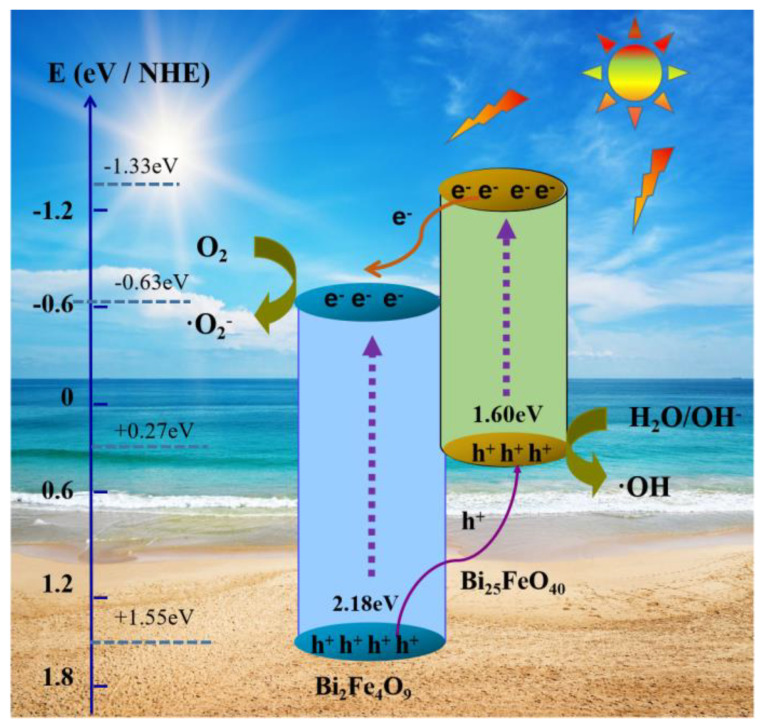
The schematic illustration of photo-reactive mechanism for Bi_2_Fe_4_O_9_/Bi_25_FeO_40_ heterostructure composites.

On the basis of the above analysis, the photodegradation mechanism for the Bi_2_Fe_4_O_9_/Bi_25_FeO_40_ composites was proposed (Scheme 1). Generally, the performance of photocatalyst depends on a series of parameters, such as the surface area, adsorption capacity, light absorption, carrier recombination rate, and energy band structure [53,54,55,56,57,58]. In our case, the significant improvement of the photodegradation performance for SPS650 may be related to the following factors: firstly, due to the high-temperature sintering of the spark plasma, the surface of the modified BFO sample has more separated electrons, holes, and OH radicals. These active materials react with pollutants in the solution to show better photocatalytic activity. Secondly, the Bi_25_FeO_40_ produced by SPS treatment results in the formation of Bi_2_Fe_4_O_9_/Bi_25_FeO_40_ heterojunction. Under visible light irradiation, the photoinduced electrons tend to migrate from Bi_25_FeO_40_ to Bi_2_Fe_4_O_9_, which leads to the faster transfer and separation of photogenerated carriers at the heterojunction interface, thereby improving the photodegradation performance.

## 3. Materials and Methods

### 3.1. Synthesis

BFO powders were firstly synthesized by the hydrothermal method, as reported previously, with a little change. Typically, Bi(NO_3_)_3_∙5H_2_O and Fe(NO_3_)_3_∙9H_2_O with the molar mass ratio Bi^3+^: Fe^3+^ = 1:1 were first dissolved and stirred at 1 M HNO_3_ solution to form an aqueous solution, and pH is ~2. Then, 4 M KOH solution was slowly added into the aforementioned solution with vigorously stirring for 1 h to tune the pH value to 9 (here, HNO_3_ was used as dissolvent, and KOH as precipitator and mineralizer). After that, it was quickly transferred into a teflon-lined stainless-steel autoclave and heated at 200 °C for 12 h. Finally, the product was collected and washed with deionized water and alcohol several times to about pH 7, followed by drying at 80 °C overnight. According to the XRD analysis (Figure 1), the obtained sample belonged to orthorhombic Bi_2_Fe_4_O_9_ (JCPDS 25-0090) and was named BFO.

Next, the obtained BFO sample was further treated using the SPS technology. In a typical SPS heat-treatment process, 1.0 g of the as-prepared BFO powder was first put into a cylindrical carbon die with an inner diameter of 15 mm and then transferred into the SPS device (SPS-3.20 MKII, Sumitomo Coal Mining Co. Ltd., Tokyo, Japan) for sintering. The temperature was raised to the set temperature with a heating rate of 50 °C/min and kept at the temperature for 5 min under 60 MPa, then naturally cooled to room temperature. During the sintering process, the sample temperature was measured using an infrared camera through a hole in the middle of the cylindrical carbon die. The modified BFO sample after SPS heat treatment were denoted as SPSX, and X (X = 300–700) represents the set temperature from 300 °C to 700 °C.

### 3.2. Characterization

The phase composition of the samples was analyzed by Bruker D8 X-ray diffractometer (XRD, Cu Kα, λ = 1.5406 Å), the sampling interval was 0.02°, the sampling rate was 5 °/min, and the scanning range of the sample was 10°–70°. TEM (TEM, JEOL JEM-2010 FEF, Japan) was used to further confirm the morphology of the obtained samples. Ultraviolet-visible diffuse reflectance spectroscopy (UV–Vis DRS) analysis adopted U-4100 solid spectrophotometer, and the test wavelength range was 200–800 nm. The valence bands of the sample surfaces were characterized using X-ray photoelectron spectroscopy (XPS, Thermo Scientific Escalab 250, Waltham, MA, USA) with a monochromatic Al K X-ray source. All binding energies were referenced to the C 1s peak (284.6 eV) arising from adventitious carbon. The specific surface areas of the catalysts were determined by applying the Brunauer–Emmett–Teller (BET) method to the adsorption of nitrogen at 77 K. All the samples were degassed at 180 °C prior to nitrogen adsorption measurements. The photocurrents, electrochemical impedance spectroscopy (EIS), and Mott–Schottky were measured by an electrochemical analyzer (CHI660A, CH Instruments Co. Shanghai, China) at room temperature. The photoluminescence (PL) spectroscopy was carried out with the FLs980 full-function steady-state/transient fluorescence spectrometer in Edinburgh, UK, with an excitation wavelength of 325 nm. Total organic carbon (TOC) was measured by a Multi N/C 3100 TOC analysis instrument.

#### Photocatalytic Performance

The photocatalytic performance was measured by degrading the 10 mg/L tetracycline hydrochloride (TCH) aqueous solution under visible light. Typically, 50 mg of catalyst was added to 50 mL of TCH solution and kept in the dark for 1 h to reach adsorption-desorption equilibrium. After that, a 350 W xenon lamp and 420 nm cut-off filter were used as the light source to simulate sunlight, and samples were taken every 20 min.

## 4. Conclusions

In summary, the self-doped Bi_2_Fe_4_O_9_/Bi_25_FeO_40_ heterostructure composites were successfully obtained by spark plasma sintering technology, combined with a facile wet chemical process. The ratio of Bi_2_Fe_4_O_9_ to Bi_25_FeO_40_ in the composites was controlled by the spark plasma sintering temperature. The as-prepared sample (SPS650) exhibited significantly higher photoelectric performance and photocatalytic activity than that of Bi_2_Fe_4_O_9_ and Bi_25_FeO_40_ on the degradation of TCH. It was found that formation of well-defined heterojunction between Bi_2_Fe_4_O_9_ and Bi_25_FeO_40_, which effectively sped the transformation and separation of photoinduced carriers, correspondingly resulted in the enhancement of the photoelectric property and photocatalytic activity. Anyway, it could be easily recycled without an obvious decrease of photocatalytic activity. This study provides a simple and economic method for the facile and quick synthesis of a highly active bismuth ferrite-based visible-light-driven photocatalyst for practical applications.

## Data Availability

Not applicable.

## Supplementary Materials

The following supporting information can be downloaded at: https://www.mdpi.com/article/10.3390/ijms232012652/s1.
