# Supplementary material for: Spark Plasma Sintering-Assisted Synthesis of Bi2Fe4O9/Bi25FeO40 Heterostructures with Enhanced Photocatalytic Activity for Removal of Antibiotics
Source: Int J Mol Sci. 2022 Oct 21;23(20):12652. doi: 10.3390/ijms232012652 (PMC9604494; doi:10.3390/ijms232012652)
Supplement: Supplementary file 1 [file ijms-23-12652-s001.zip › ijms-1934950-supplementary.pdf]

Supporting Information

Spark Plasma Sintering-Assisted Synthesis of $Bi_2Fe_4O_9/Bi_{25}FeO_{40}$ Heterostructures with Enhanced Photocatalytic Activity for Removal of Antibiotics

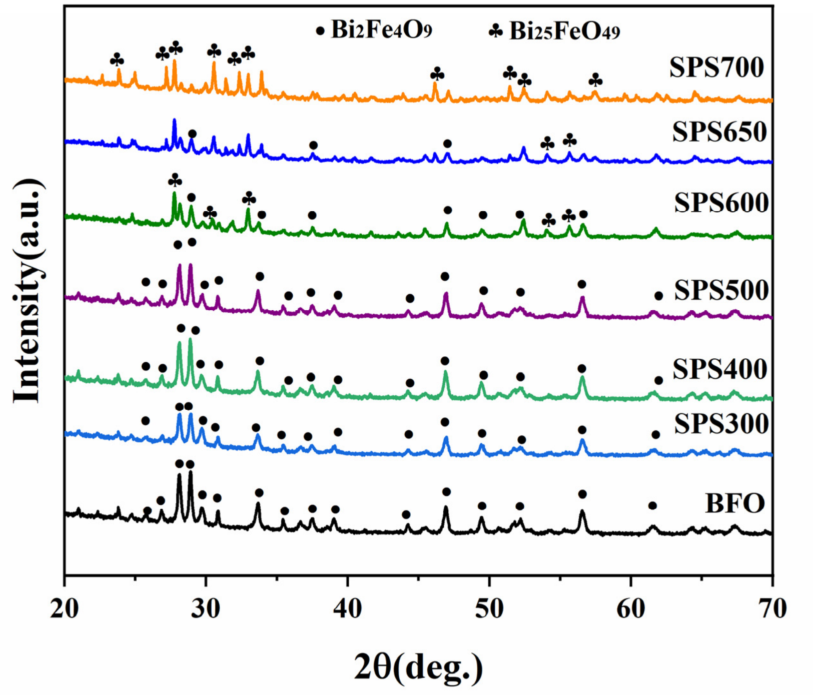

Figure S1. X-ray diffraction (XRD) spectra of as-prepared samples.

Table S1. The Percentage of $Bi_2Fe_4O_9$ and $Bi_{25}FeO_{40}$ in the BFO heterojunction composites.

	BFO	SPS600	SPS650	SPS700
$Bi_2Fe_4O_9$	100%	76.3%	51.4%	5.5%
$Bi_{25}FeO_{40}$	0%	23.7%	48.6%	94.5%

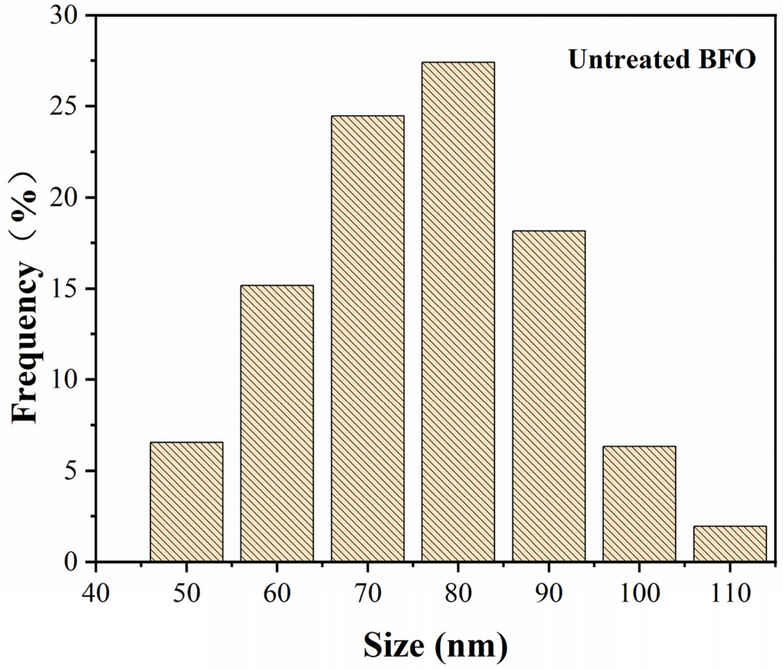

Figure S2. Size distribution of untreated BFO.

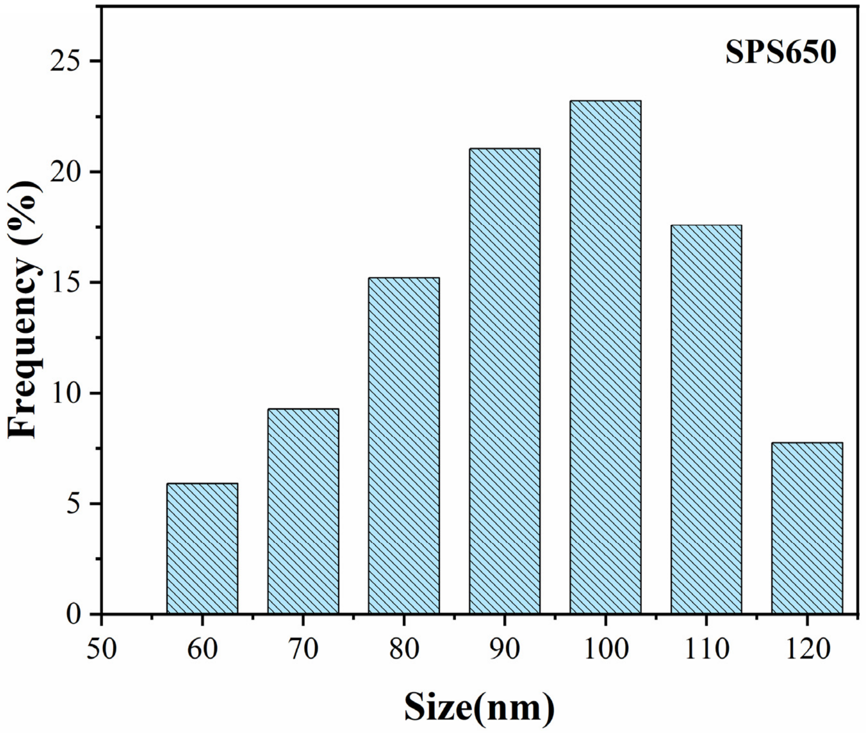

Figure S3. Size distribution of SPS650.

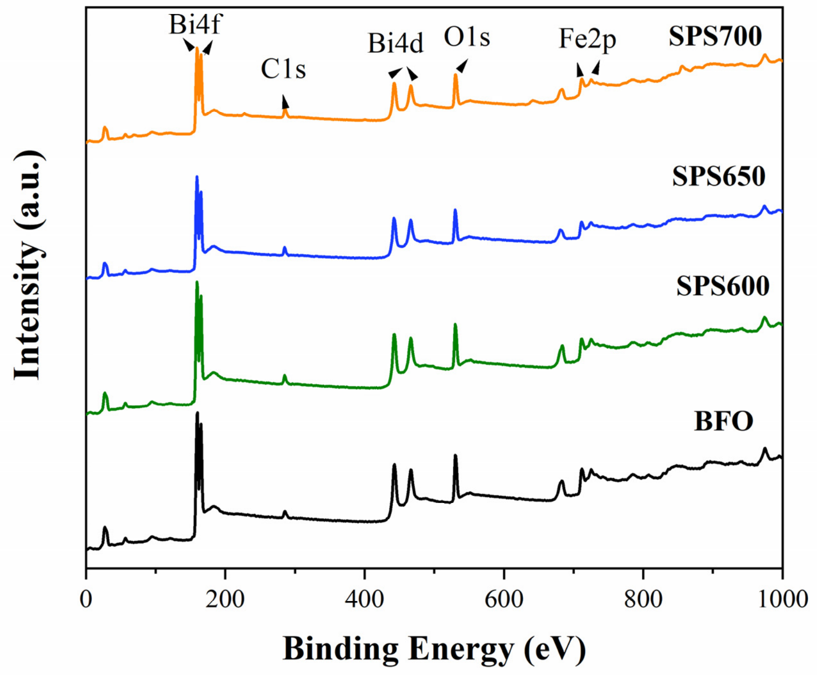

Figure S4. survey XPS spectra of different samples.

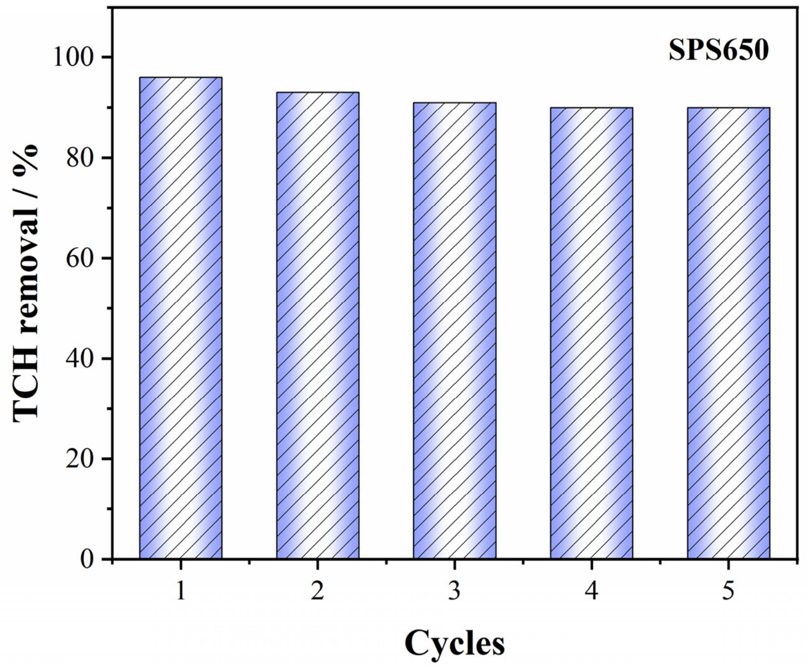

Figure S5. Cycling stability of SPS650 for TCH degradation.

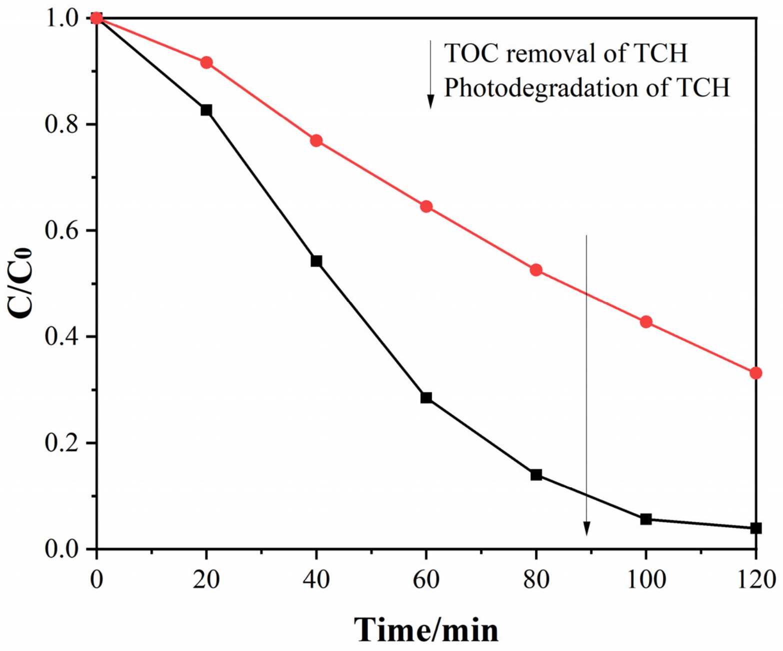

Figure S6. The mineralization ability of SPS650 sample for the TCH in terms of TOC removal.

Table S2. Calculated Apparent Quantum Yield (AQY).

Wavelength (nm)	Degraded molecular s (μmol)	Light intensity (mW cm^{-2})	Irradiation area (cm^2)	Irradiation time (h)	AQY (%)
420	16.7	7.80	1.67	2	4.66

The number of incident photons:

$$N = \frac{E\lambda}{hc} = \frac{8.5 \times 1.67 \times 10^{-3} \times 2 \times 3600 \times 420 \times 10^{-9}}{6.626 \times 10^{-34} \times 3 \times 10^{8}} = 2.155 \times 10^{20}$$

$$AQY\% = \frac{\text{the number of degraded moleculars}}{N} \times 100\%$$

$$= \frac{6.02 \times 10^{23} \times 1.67 \times 10^{-6}}{2.155 \times 10^{20}} \times 100\%$$

$$= 4.66\%$$

Table S3. Comparison of catalytic activities of different catalysts.

Catalyst	Reaction conditions	Kinetics	References
$Bi_2Fe_4O_9$@$Bi_{25}FeO_{40}$	Catalyst = 0.2 g/L, [TC] = 50 mg/L,	Pseudo first order kinetic $k = 2.5 \times 10^{-2}$ min^{-1}	This work
Schorl	Catalyst = 10.0 g/L, [TC] = 100 mg/L, [H_2O_2] = 9.9 mM, pH = 3.0, 40 °C	Pseudo first order kinetic $k = 0.70 \times 10^{-2}$ min^{-1}	[1]
Carbon dots/MoO_3/g-C_3N_4	Catalyst = 0.6 g/L, [TC] = 20 mg/L,	Pseudo first order kinetic $k = 2.31 \times 10^{-2}$ min^{-1}	[2]
Ag/Ag_2CO_3/$BiVO_4$	Catalyst = 0.4 g/L, [TC] = 20 mg/L,	Pseudo first order kinetic $k = 1.86 \times 10^{-2}$ min^{-1}	[3]
Ag/$AgIn_5S_8$	Catalyst = 0.3 g/L, [TC] = 10 mg/L, pH = 4.4	Pseudo first order kinetic $k = 2.30 \times 10^{-2}$ min^{-1}	[4]
Fe_3O_4/rGO/TiO_2	Catalyst = none, [TC] = 20 mg/L, [H_2O_2] = 20 mM, pH = 3	Not mentioned	[5]
Chalcopyrite	Catalyst = 1 g/L, [TC] = 89 mg/L, pH = 5.94	Pseudo first order kinetic $k = 0.91 \times 10^{-2}$ min^{-1}	[6]

Reference

1. Zhang, Y.; Shi, J.; Xu, Z.; Chen, Y.; Song, D. Degradation of tetracycline in a schorl/H_2O_2 system: Proposed mechanism and intermediates. *Chemosphere* 2018, 202, 661–668.
2. Xie, Z.; Feng, Y.; Wang, F.; Chen, D.; Zhang, Q.; Zeng, Y.; Lv, W.; Liu, G. Construction of carbon dots modified MoO_3/g-C_3N_4 Z-scheme photocatalyst with enhanced visible-light photocatalytic activity for the degradation of tetracycline. *Appl. Catal. B Environ.* **2018**, *229*, 96–104.
3. Liu, Y.; Kong, J.; Yuan, J.; Zhao, W.; Zhu, X.; Sun, C.; Xie, J. Enhanced photocatalytic activity over flower-like sphere Ag/Ag_2CO_3/$BiVO_4$ plasmonic heterojunction photocatalyst for tetracycline degradation. *Chem. Eng. J.* **2018**, *331*, 242–254.
4. Deng, F.; Zhao, L.; Luo, X.; Luo, S.; Dionysiou, D. D. Highly efficient visible-light photocatalytic performance of Ag/AgIn5S8 for degradation of tetracycline hydrochloride and treatment of real pharmaceutical industry wastewater. *Chem. Eng. J.* **2018,** *333*, 423–433.
5. Wang, W.; Xiao, K.; Zhu, L.; Yin, Y.; Wang, Z. Graphene oxide supported titanium dioxide & ferroferric oxide hybrid, a magnetically separable photocatalyst with enhanced photocatalytic activity for tetracycline hydrochloride degradation. *RSC Adv.* **2017**, *7*, 21287–21297.
6. Barhoumi, H.; Olvera-Vargas, N.; Oturan, D.; Huguenot, A.; Gadri, S.; Ammar, E.; Brillas, M.A. Kinetics of oxidative degradation/mineralization pathways of the antibiotic tetracycline by the novel heterogeneous electro-Fenton process with solid catalyst chalcopyrite. *Appl. Catal. B Environ.* **2017**, *209*, 637–647.
